# An Unusual Cause of Dyspnea and Thoracic Pressure

**DOI:** 10.1155/2019/2574858

**Published:** 2019-10-20

**Authors:** Claire Chakkalakal, Rezo Jorbenadze, Meinrad Gawaz

**Affiliations:** University Hospital, Department of Cardiology and Cardiovascular Medicine, University of Tübingen, 72076 Tübingen, Germany

## Abstract

There is a high prevalence of hepatic cysts in the general population. Simple cysts are most of the times asymptomatic and are usually detected incidentally on ultrasonography, computed tomography, or magnetic resonance imaging. Symptoms may range from abdominal discomfort and pain, early satiety, dyspepsia, nausea, and vomiting to jaundice and portal hypertension due to obstruction of adjacent structures. Complications include spontaneous hemorrhage, infection, thrombosis, and atrophy of surrounding hepatic tissue. We present a unique case of a middle-aged patient with acute onset of dyspnea and thoracic pressure due to compression of the right ventricle by a large hepatic cyst.

## 1. Introduction

Nonparasitic hepatic cysts occur in approximately 5% of the global population [1]. Since most hepatic cysts remain asymptomatic, the exact number is unknown. Liver cysts are fluid-filled cavities and normally require no treatment. The symptoms are strongly variable and usually depend on the cysts' size, location, type, and number. Most commonly described symptoms are abdominal pain or discomfort, early satiety, and nausea. In some cases, obstructive jaundice or portal hypertension may occur due to compression of adjacent structures. Hypotension and thrombosis due to atypical vena cava-compression syndrome are rare. Complications include spontaneous cyst rupture, hemorrhage into the abdominal cavity, and infections. Besides symptomatic treatment, therapeutic options range from percutaneous drainage and open or laparoscopic deroofing to hepatic resection [1]. We present a rare case of a middle-aged female patient with sudden onset of cardiopulmonary symptoms due to a large simple cyst of the liver.

## 2. Case Presentation

A 51-year-old woman presented herself in our emergency room (ER) with dyspnea and retrosternal thoracic pressure. The symptoms had started 2 years earlier, with a distinct deterioration over the course of the 4 weeks preceding her admission. According to the patient, the symptoms had culminated in acute shortness of breath and tightness of the chest, accompanied by nocturnal panic attacks, dyspepsia, and general weakness 2 nights before. The symptoms primarily occurred when lying down. There were no cardiovascular risk factors except for a positive family history for aortic aneurysms. Except for a hysterectomy many years earlier, her medical history was empty. The electrocardiograms (ECGs) recorded at her general practitioner (GP) during the past few years and, in particular, during the weeks prior to her admission, were all normal. A physical stress test had been performed at a local cardiologist with no conclusive results regarding exercise-induced alteration in repolarization or arrhythmias. Two weeks before, she had been referred to a local radiological center for computed tomography (CT) of the chest in order to detect potential lung embolism or aortic aneurysms. In regard to her family history, this seemed to be a relevant differential diagnosis, but the scan remained without noticeable mediastinal findings.

Upon admission to our ER, the initial ECG showed no abnormalities, blood pressure was 130/70 mmHg, oxygen saturation was between 97 and 100%. Her lab results were all normal. With troponin, CK, and D-dimers within physiological range, there were again no indicators for lung embolism, aortic aneurysm, or myocardial infarction. Furthermore, no infection or other pathological findings like elevated liver or kidney values were detected in the blood test results.

Echocardiography showed a good left-ventricular function and no valve dysfunctions. However, in the four- and five-chamber view, the right ventricle appeared to be compressed from the outside. The subxiphoid view confirmed this. The structure compressing the right side of the heart proved to be a large cystic lesion of the liver. In the consecutive ultrasound of the abdomen, the cyst measured 9 × 7 cm. Its size and subdiaphragmatic location left no doubt that it caused the symptoms described by the patient, in the absence of any other pathological findings.

She was referred to abdominal surgery and after negative echinococcus test results, a laparoscopic unroofing was scheduled. Several days before the appointment, the patient was readmitted due to increasing shortness of breath. The ultrasound showed that the cyst's total dimensions were 10.7 × 8.3 cm now and thus had grown over 1 cm per side in only 3 weeks. This fast progress explains why the affection appeared as a sudden onset, although the symptoms had started 2 years earlier.

**Figure 1:**
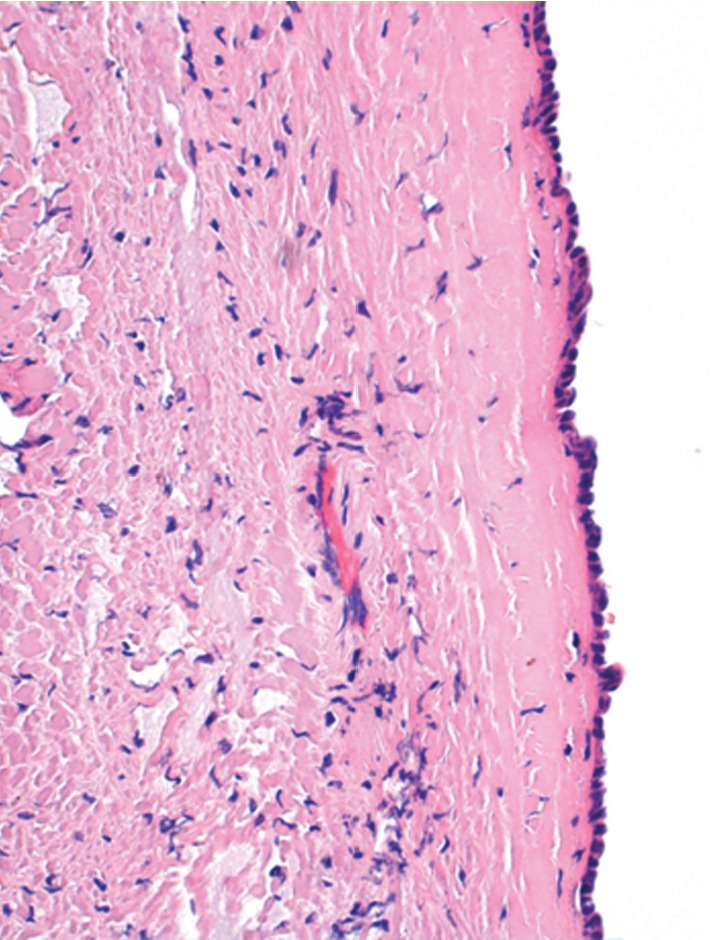
Cystic lesion with thin collagenous wall, lined by biliary type epithelium (flat to cuboidal). No pathohistological sign of inflammation or infection.

Laparoscopic cystectomy was performed without complications. The symptoms previously described ceased abruptly afterwards. The histopathological findings gave no evidence of infection or inflammatory processes (Figure 1).

## 3. Discussion

Simple cysts account for the majority of hepatic cysts. Less frequent and more complicative cystic lesions include echinococcosis, cystadenoma, and cystadenocarcinoma [2]. In regard to the relatively high prevalence of hepatic cysts in general, symptoms or complications are rare. The most frequent ones are abdominal discomfort, nausea, and other gastrointestinal symptoms [1].

**Figure 2:**
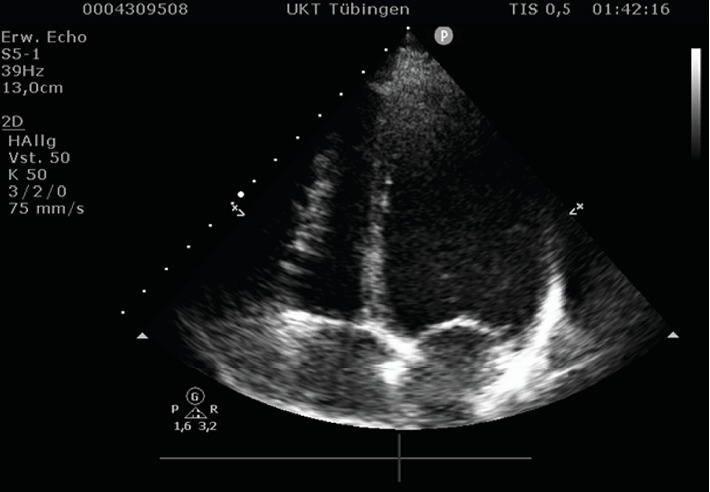
Echocardiogram, four-chamber view: extrinsic compression of the right ventricle.

**Figure 3:**
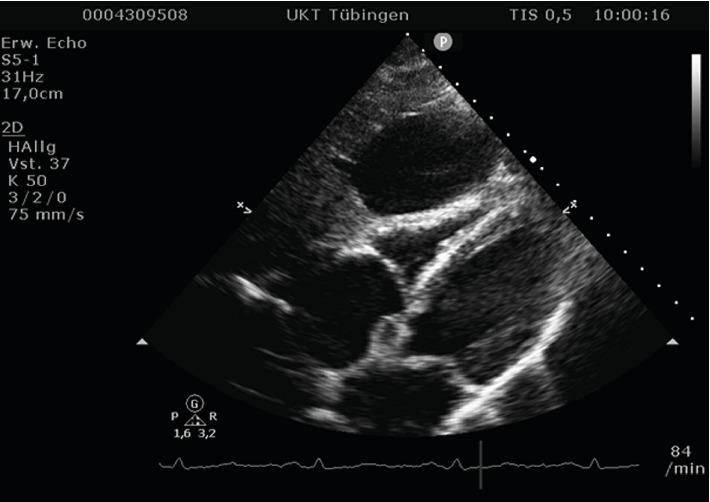
Echocardiogram, subxiphoidal view: cystic lesion of the liver compressing the right ventricle.

Cyst rupture and hemorrhage as well as infections account for the most common complications. Cardiopulmonary symptoms or complications are an exception and usually manifest themselves in form of hypotension and thrombosis. Acute severe retrosternal pressure or thoracic tightness and respiratory symptoms, such as dyspnea, are rather typical for cardiac or cardiopulmonary conditions, the most common ones being myocardial infarction, heart failure, aortic aneurysms, and lung embolism. Similar symptoms have been described to occur in pericardial cysts [3, 4] due to hemodynamic alterations and compression of the right ventricle as was also presented in this case (Figures 2 and 3). Isolated compression of the right heart chambers has also occurred in a few cases of patients with pectus excavatum, who showed comparable symptoms, such as dyspnea and thoracic pain or tightness [5–7].

This is the first reported case in which an abdominal cyst was compromising organs of the adjacent cavity to such an extent that it manifested itself as acute cardiopulmonary condition.

**Figure 4:**
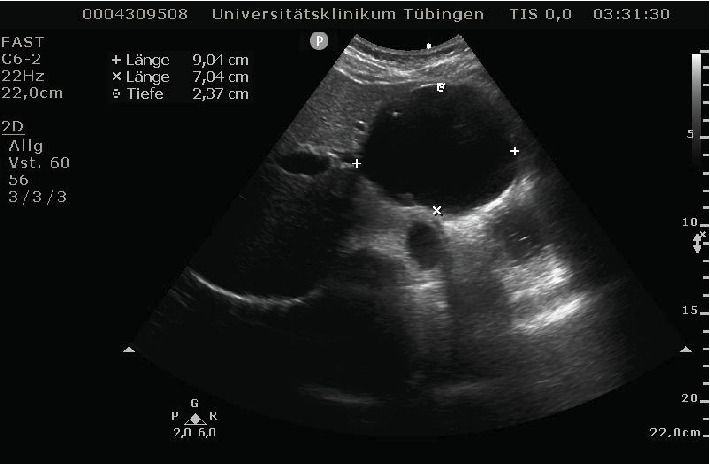
Sonography of the abdomen showing a cystic lesion in the liver (dimensions: 9 × 7 cm).

Since simple hepatic cysts are usually asymptomatic, the diagnosis is mostly incidental. They range from a few millimeters to enormous sizes, and the predominant location is the right hepatic lobe [2]. Laboratory results are usually normal. The cysts can be detected by sonography, CT, or magnetic resonance imaging [8]. On ultrasound imaging, they appear as spherical or oval fluid-filled, nonseptated cavities with sharp and smooth borders (Figure 4). These distinct sonographic features combined with the lack of dorsal shadowing distinguish them from potential harmful cystic lesions, such as the aforementioned echinococcosis, cystadenoma, and cystadenocarcinoma [8].

Exclusion of other differential diagnoses with cystic appearance on radiographic imaging such as hepatic abscess, hemangioma, hamartoma, and even necrotic malignant neoplasms can usually be made based upon a combination of sonographic findings, clinical presentation, and lab results [2, 8].

Treatment modalities of hepatic cysts in general range from mere observation and drainage to surgical measures such as laparoscopic or open unroofing and hepatic resection. The choice of procedure mainly depends on the type of cyst, size, and symptoms [1, 8]. The general therapeutic recommendation for simple but symptomatic hepatic cysts is wide unroofing, either by open or laparoscopic surgery [9], as was also performed in this case, due to the minimal recurrence rates of simple cysts after surgical treatment.

**Figure 5:**
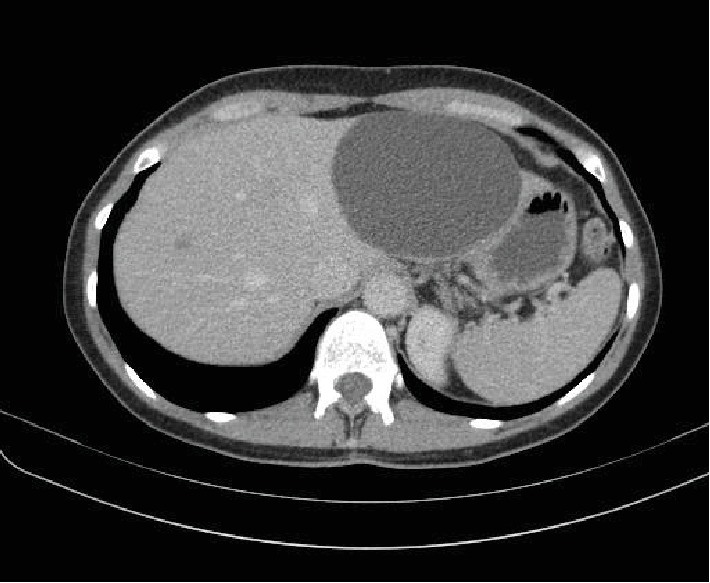
CT scan: large hepatic cyst in the left hepatic lobe.

In this unique case, although neighboring abdominal structures, including the left-lobular cyst, are clearly visible on the CT scan (Figure 5), it has to be emphasized that the cyst's impact on the circulatory and cardiopulmonary system could not be determined by CT scan. This is due to the fact that a CT scan with focus on pulmonary embolism does not show the dynamics and alterations of the heart and potential impact on hemodynamics during an entire heart cycle.

With this case report, we presented an unusual cause of dyspnea and thoracic pressure. Although there were no evident alterations in preload or pulmonary pressure, the almost complete extrinsic compression of the right ventricle is the only explanation for the cardiorespiratory symptoms described by the patient. Right ventricular compression only became visible when performing an echocardiography, which also underlines its value as a radiation-free, economic, and convenient diagnostic tool in daily clinical practice.
